# Osteoclast Differentiation Is Impaired in a Subgroup of SLE Patients and Correlates Inversely with Mycophenolate Mofetil Treatment

**DOI:** 10.3390/ijms160818825

**Published:** 2015-08-12

**Authors:** Barbara G. Fürnrohr, Benjamin Rhodes, Luis E. Munoz, Katrin Weiß, Tim J. Vyse, Georg Schett

**Affiliations:** 1 Department of Internal Medicine 3 and Institute for Clinical Immunology, Ulmenweg 18, University of Erlangen-Nuremberg, 91054 Erlangen, Germany; E-Mails: luis.munoz@uk-erlangen.de (L.E.M.); kweiss@molmed.uni-erlangen.de (K.W.); georg.schett@uk-erlangen.de (G.S.); 2 Division of Genetic Epidemiology and Division of Biological Chemistry, Innrain 80/IV, Medical University Innsbruck, 6020 Innsbruck, Austria; 3 Department of Rheumatology, University Hospitals Birmingham NHS foundation trust, Edgbaston, B15 2GW Birmingham, UK; E-Mail: benjamin.rhodes@uhb.nhs.uk; 4 Division of Genetics and Molecular Medicine and Division of Immunology, Infection and Inflammatory Disease, King’s College London, Great Maze Pond, SE1 9RT London, UK; E-Mail: timothy.vyse@kcl.ac.uk; 5 Division of Molecular Immunology of the Department of Internal Medicine 3, Ulmenweg 18, University of Erlangen-Nuremberg, 91054 Erlangen, Germany

**Keywords:** osteoclastogenesis, mycophenolate mofetil, interferon alpha, systemic lupus erythematosus, RANKL

## Abstract

Osteoporosis can arise in systemic lupus erythematosus (SLE) patients secondary to medication and/or chronic inflammation. To analyze if patients with SLE have phenotypically-impaired osteoclastogenesis, we differentiated *ex vivo* monocytes from 72 SLE patients and 15 healthy individuals into osteoclasts followed by TRAP staining and counting. We identified a subgroup of SLE patients (45%) with a significantly impaired osteoclast differentiation, relative to the other SLE patients or healthy individuals (OR 11.2; 95% CI 1.4–89.9). A review of medication indicated that patients with osteoclast counts equal to healthy donors were significantly more likely to be treated with mycophenolate mofetil (MMF) compared to patients with impaired osteoclastogenesis. We analyzed expression of RANKL and the MMF target genes *IMPDH1* and *IMPDH2* in osteoclasts by qPCR, but detected no difference. Since MMF might influence interferon-α (IFNα) and -γ (IFNγ) we measured serum IFNα and IFNγ levels. Patients with very low osteoclast counts also had comparably higher IFNα serum levels than patients with normal osteoclast counts. We conclude that *in vitro* osteoclastogenesis is impaired in a subgroup of SLE patients. This correlates inversely with MMF treatment and high IFNα serum levels. Further observational study will be required to determine whether this translates into a clinically meaningful effect.

## 1. Introduction

Although osteoporosis is not a diagnostic feature of systemic lupus erythematosus (SLE), it commonly occurs secondary to medication, particularly long-term glucocorticoids, and/or as a consequence of chronic inflammation. In addition, 90% of SLE patients are female, a group already at comparably higher risk for osteoporosis.

The differentiation, metabolism, growth and remodeling of bone is tightly regulated and balanced by osteoblasts, chondrocytes and osteoclasts. Osteoporosis occurs when this remodeling cycle gets unbalanced, with either increased osteoclast activity and/or reduced osteoblast function. Osteoblasts are derived from mesenchymal cells, whereas osteoclasts are derived from the mononuclear hematopoietic myeloid cell lineage and circulate as precursors through the blood stream. Differentiation of hematopoietic precursors into monocytes/macrophages has been shown to be intrinsically impaired in patients with SLE [1]. Crucial for osteoclast formation and regulation are the cytokines macrophage-colony-stimulating factor (M-CSF) and receptor activator of NF-κB ligand (RANKL), which are secreted by various cell types including osteoblasts [2].

A number of the drugs used to treat SLE, such as glucocorticoids, cytostatic agents and cyclosporin are known to mediate secondary osteoporosis. The immunosuppressant mycophenolate mofetil MMF has become a valuable drug for the treatment of SLE, especially for treatment of lupus nephritis. MMF has no known effect on bone remodeling in SLE; indeed in a recent study of *ex vivo* osteoclast differentiation of cells derived from patients after renal transplantation MMF, in contrast to sirolimus, had no effect on osteoclast differentiation [3]. MMF is a potent, selective, non-competitive inhibitor of the *de novo* pathway of purine synthesis, thereby mainly affecting activated B and T lymphocytes during proliferation [4]. MMF has also been shown to affect other cell types including monocytes [5] and podocytes [6]. MMF seems to down-regulate important monocytic adhesion molecules thereby inhibiting monocyte adhesion to endothelial cells [5]. Given this effect on cells of myeloid lineage we were interested to study osteoclast differentiation in SLE, in particular in patients treated with MMF.

In this study we identified a subgroup of SLE patients with very low *in vitro* osteoclast counts. Surprisingly in this subgroup fewer patients were receiving MMF therapy compared to patients with an osteoclast count equal to healthy individuals.

## 2. Results and Discussion

### 2.1. Results

#### 2.1.1. *In Vitro* Monocyte-Derived Osteoclast Differentiation of SLE Patients and Controls

**Figure 1:**
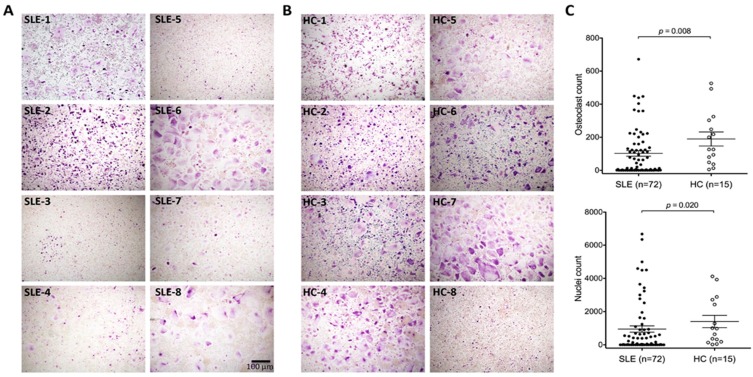
*In vitro* osteoclastogenesis is impaired in SLE patients. An example of images of TRAP stained osteoclasts for eight different (**A**) patients with systemic lupus erythematosus (SLE) and (**B**) normal healthy controls (HC); (**C**) Graphs show the osteoclasts and nuclei count out of three different wells for SLE patients and healthy controls. *p*-values were calculated by Mann-Whitney *U* test.

In this study we compared the *ex vivo* osteoclast differentiation capacities of monocytes derived from 72 individual SLE patients and 15 healthy controls (HC). Examples of acquired images of differentiated osteoclasts from patients and controls are depicted in Figure 1A,B. Images were taken in triplicate from differentiated osteoclasts of each donor to count stained osteoclasts. There was a significant reduction in osteoclasts in SLE patients compared to HC (χ^2^ = 7.53, *p* = 0.0061). In particular, we observed a subgroup of patients (45%) that showed markedly reduced (overall count less than 10 osteoclasts) or even completely impaired osteoclastogenesis. A similar result was obtained by counting nuclei to distinguish multinucleate cells (Figure 1C). When we excluded this subgroup with a marked osteoclast differentiation defect from the analysis, osteoclast numbers between the remaining SLE patients and HC were similar.

#### 2.1.2. Osteoclast Differentiation and MMF, IFNα and Corticosteroids

**Figure 2:**
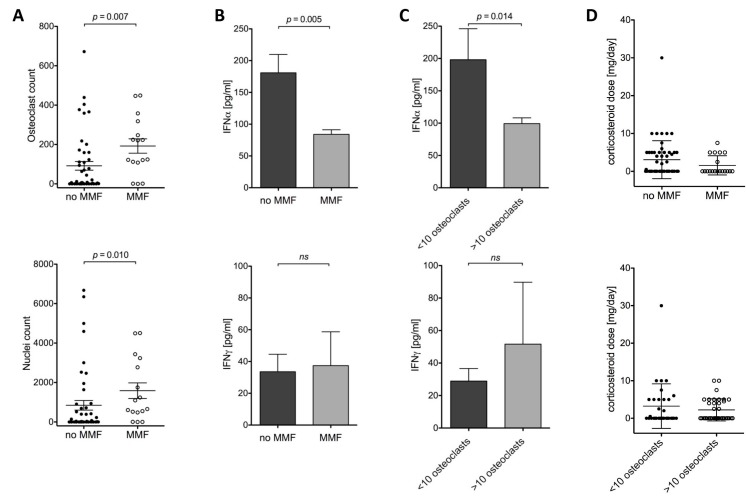
SLE patients without MMF therapy have a lower osteoclast count and higher IFNα levels in serum. (**A**) Patients with SLE with mycophenolate mofetil (MMF) treatment have significantly higher numbers of osteoclasts and nuclei generated *in vitro* compared to patients without MMF; (**B**) SLE patients with very low osteoclast count (<10) or without MMF treatment have higher serum IFNα levels; (**C**) No difference of IFNγ levels in plasma of SLE patients was detected; (**D**) The corticosteroid dose was not significantly different between patients with MMF or without MMF treatment or between patients with low or normal osteoclast counts; *p* Values were calculated by Mann-Whitney *U* test; ns: not significant.

We investigated if MMF therapy affected osteoclast differentiation and found that in patients who were treated with MMF at the time-point of blood withdrawal, the mean osteoclast count was comparable to healthy controls. However, patients lacking MMF administration showed considerable lower osteoclast and nuclei count (Figure 2A). Since MMF is considered to primarily act by affecting lymphocyte activity rather than myeloid cell function, we considered an indirect effect of MMF on *in vitro* osteoclast differentiation to be more likely than a direct effect. Therefore, we analyzed serum samples of SLE patients with and without MMF treatment for interferon-α (IFNα) and interferon-γ (IFNγ) by ELISA. Patients under MMF treatment had significantly lower IFNα plasma levels compared to patients without MMF treatment. There was no difference in IFNγ levels (Figure 2B). Consistently, when analyzing patients according to their osteoclast count, elevated IFNα levels were also observed in patients with osteoclast differentiation defect, with no difference for IFNγ (Figure 2C). When we analyzed corticosteroid doses in MMF treated or untreated patients we could not detect a significant difference between both groups. Similarly, corticosteroid treatment or dose did not seem to affect *in vitro* osteoclast differentiation (Figure 2D). Independent of the corticosteroid dose, the percentage of patients treated with corticosteroids was similar in both groups, with 44% for low osteoclast count and 43% for normal osteoclast differentiation.

#### 2.1.3. Effect of MMF on IMPDH1, IMPDH2 and RANK mRNA Expression

**Figure 3:**
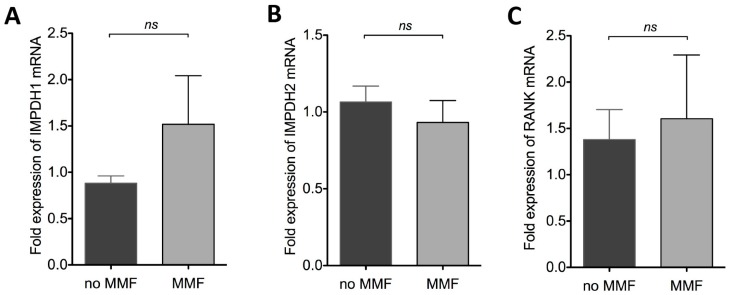
MMF therapy does not affect mRNA expression of IMPDH1, IMPDH2, and RANK in differentiated osteoclasts. mRNA was extracted from differentiated osteoclasts from SLE patients with (*n* = 5) and without MMF (*n* = 15) therapy. cDNA synthesis was carried out, and qPCR analysis of (**A**) IMPDH1, (**B**) IMPDH2 and (**C**) RANK was performed using SYBR Green and quantified by ΔΔ*C*_t_ method, normalized to two different housekeeping genes RPL27 and GAPDH. ns: not significant.

MMF is a reversible inhibitor of inosine monophosphate dehydrogenase (IMPDH), an enzyme involved in purine synthesis. The IMPDH protein family consists of two isoforms, namely IMPDH1, which is constitutively expressed, and the inducible isoform IMPDH2 [7,8]. To analyze if MMF might have an impact on IMPDH expression in differentiated osteoclasts, we performed qPCR analysis for *IMPDH1* and *IMPDH2*. We observed an induction of *IMPDH1* mRNA in patients under MMF treatment; however, this increase was not significant (Figure 3A). No difference in expression was observed for *IMPDH2* (Figure 3B). Since RANKL is crucial for osteoclast differentiation, we wanted to rule out that low osteoclast differentiation is due to reduced RANK expression. At least on the mRNA level there was no difference in RANK expression when comparing patients with or without MMF (Figure 3C).

#### 2.1.4. SLE Disease Activity Was Similar Irrespective of Osteoclast Differentiation or MMF Treatment

**Figure 4:**
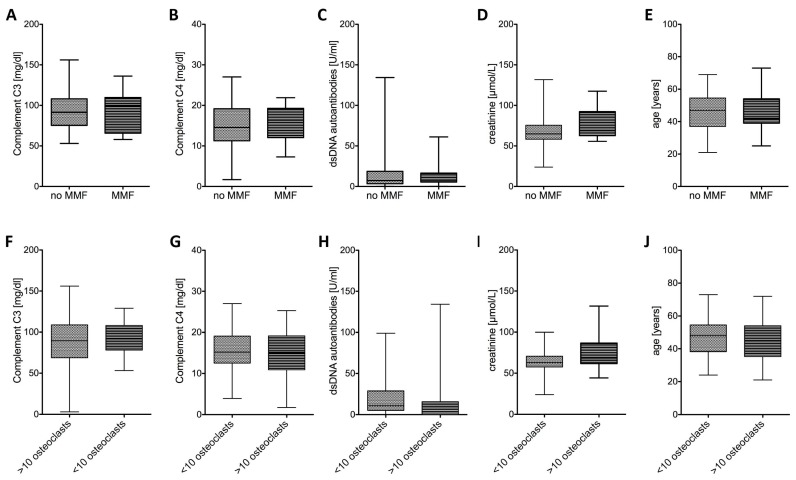
Disease activity was comparable for patients with or without MMF treatment. Disease activity status was similar in SLE patients who were under MMF medication as measured by (**A**) complement C3, (**B**) complement C4, and (**C**) dsDNA autoantibodies. Patients with low osteoclast counts had comparable (**F**) C3, (**G**) C4, and (**H**) dsDNA autoantibody titers. (**D**,**I**) Creatinine levels were also similar in both groups. Average age was similar in (**E**) MMF treated or untreated patients and in patients with (**J**) low or normal osteoclast count.

Complement C3, C4, and dsDNA autoantibody titers were used to evaluate the disease activity status of SLE patients and to compare MMF treated with MMF-untreated patients. There was no significant difference between both groups of patients (Figure 4A–C). Furthermore, when we analyzed patients according to their osteoclast count profile C3, C4, and dsDNA antibody titers were similar in both groups (Figure 4F–H). Of our study group no patient had advanced renal disease. To further analyze the renal function we used serum creatinine levels. MMF treatment did not severely affect creatinine levels in SLE patients (Figure 4D). Patients with low osteoclast numbers had marginally lower levels of creatinine, when compared to patients with normal osteoclast counts (Figure 4I). Therefore, we conclude that renal function was not impaired in patients with low osteoclast count. Since age is known to affect osteoporosis, we have additionally analyzed the age of SLE patients between groups, but could not detect a significant difference (Figure 4E,J).

#### 2.1.5. MMF and Full Blood Count

**Figure 5:**
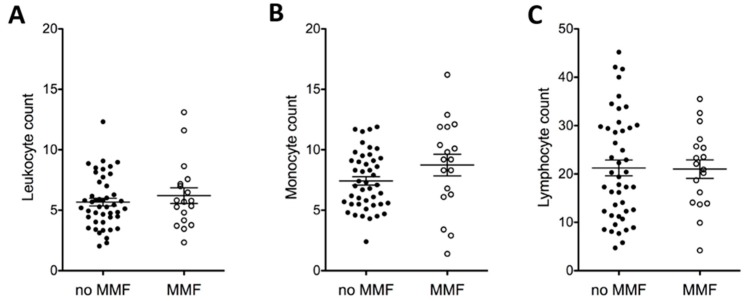
MMF does not directly affect full blood count. (**A**) Leukocyte, (**B**) monocyte, and (**C**) lymphocyte counts were acquired from patient records and plotted in dependency of MMF administration to SLE patients.

To ensure that the differences in osteoclast numbers we observed were not simply due to differences in the number of monocyte precursors from the patients treated with MMF we plotted the leukocyte, monocyte, and lymphocyte counts dependent on MMF treatment (Figure 5). No significant difference in leukocyte, lymphocyte, or monocyte numbers was observed.

### 2.2. Discussion

In this study we differentiated *in vitro* osteoclasts from monocytes derived from SLE patients and healthy individuals and identified a subset of patients with impaired osteoclastogenesis. A significantly minor portion of SLE patients showed impaired osteoclastogenesis and were on mycophenolate mofetil (MMF) therapy compared to patients with osteoclast counts similar to healthy individuals, who were more frequently treated with MMF and who had lower IFNα serum levels. Whether this effect of MMF on osteoclastogenesis is direct or indirect remains to be elucidated. We can rule out that disease activity measured by complement C3, C4, and dsDNA antibodies affect osteoclastogenesis, since patients with low or normal osteoclast counts had similar titers. Likewise creatinine levels were comparable between groups. Furthermore, we could not detect any significant difference in patients with or without MMF treatment for corticosteroids, C3, C4, dsDNA antibodies or creatinine.

Mycophenolic acid is the active agent of MMF and acts as potent, selective and reversible inhibitor of two target genes of the inosine monophosphate dehydrogenase (IMPDH) family, namely IMPDH1 and IMPDH2. Both enzymes convert inosine monophosphate to guanosine monophosphate, which is essential and rate limiting in purine biosynthesis of T and B cells, therefore crucial for B and T cell growth [4]. IMPDH1 is expressed mainly in resting lymphocytes compared to IMPDH2, which is expressed rather in stimulated, proliferating lymphocytes [9]. Nevertheless, MMF does not solely affect lymphocyte function but can also alter the activity of other immune cells. The reason why MMF controls lymphocyte more than phagocyte activity is the fact that phagocytic cells are not essentially dependent on IMPDH, since they have access to an alternative purine source in recycling of cleared cellular material [10]. Nonetheless, as has been shown by others, monocytes do indeed express IMPDH1, which is even preferentially induced in monocytes rather than lymphocytes after prolonged MMF treatment [11]. Consistent with these observations, our data revealed a trend towards higher expression of IMPDH1 mRNA in osteoclasts differentiated from MMF-treated SLE patients relative to patients without MMF, the difference was, however, not significant. MMF does not necessarily alter expression of target genes but primarily impair IMPDH activity. Apart from IMPDH and RANKL, MMF treatment could also target other molecules crucial for osteoclastogenesis and bone resorption, such as fibroblast growth factor-23 and mediators modulating the Wnt-signaling pathway, which was not part of this study and needs further investigation. Interestingly, we observed that MMF-treated patients with normal osteoclast counts had lower IFNα serum levels compared to patients with reduced osteoclasts and no MMF therapy. Inhibition of *in vitro* osteoclast differentiation by IFNα has already been addressed by Avent *et al.* [12] in the context of renal cell carcinoma-induced angiogenesis. The ability of MMF as a therapeutic agent for SLE to directly inhibit IFN type I production is only recently becoming a focus of investigation. Preliminary data from lupus patients suggest that MMF treatment is associated with reduction of the IFN score derived from lupus patients PBMCs [13]. In our study, we found that osteoclast differentiation was drastically reduced in a subgroup of SLE patients and this low count is especially found in patients without MMF treatment who had higher IFNα serum levels. We cannot rule out the possibility that this is due to an indirect or secondary effect, since we did not investigate the direct effect of MMF on osteoclast differentiation or IFNα levels. We did not have direct data on the Vitamin D or PTH status of these patients. We do not believe the level of renal function in these patients (Figure 4D) would make secondary hyperparathyroidism or major calcium and phosphate imbalance likely. We are aware that Vitamin D deficiency is frequently detected in patients with SLE and that this may be seasonally variable. Since our samples were randomly collected over more than one year we think it is unlikely that Vitamin D was systematically lower in a particular subset of the 72 patients analyzed in this study. Moreover we know from medication, that vitamin D was administered to five of the 72 patients with three out of these five having low, and two normal, osteoclast differentiation potential.

We would like to point out that the experiments presented in this manuscript investigated the short-term differentiation of osteoclasts from myeloid precursors. We are not presenting a comprehensive model of whole-bone function, or implying that impaired osteoclast differentiation is a surrogate marker of osteoporosis and are aware that there is currently no clinical data suggesting that MMF is osteo-protective. Further work is clearly required to delineate whether these results translate into a clinically important *in vivo* effect on osteoclastogenesis and to delineate the pathways by which MMF interacts with osteoclast differentiation pathways. This question could possibly be answered by an experiment using a murine model for lupus. Evidence from a recent study by Westenfeld *et al.*, who have analyzed therapeutic effects on osteoclast differentiation by patients with renal transplants, suggests there is no direct effect of MMF on osteoclast differentiation [3]. If this is true, then reduced osteoclast counts in SLE patients either result from MMF via silencing of the IFNα pathway, or indirectly by a yet unknown mechanism.

## 3. Experimental Section

### 3.1. Patients and Controls

A cohort of 72 unrelated well-documented German patients with SLE (66 women and six men) from the Department of Internal Medicine 3, University of Erlangen-Nuremberg, were randomly selected irrespective of stage or severity of the disease. All patients fulfilled the criteria of the American College of Rheumatology for the classification of SLE. The treatment they were receiving was entirely at the discretion of their managing clinician. A group of 15 healthy unrelated donors volunteered as controls. Sex ratio of healthy controls (HC) and SLE patients was equal and age was matched in mean. The study was approved of the ethics committee of the University of Erlangen-Nuremberg, and written informed consent was obtained from all participants.

### 3.2. Isolation of Monocytes and Osteoclast Differentiation

Peripheral blood mononuclear cells were isolated from 10 mL EDTA-blood drawn from healthy donors or SLE patients using a Ficoll gradient (Lyphoflot; BioRad, Munich, Germany). Separation of the monocyte fraction was carried out by plastic adherence to the bottom of 96-well plates (0.3 × 10^6^ cells/well) in serum-free α-MEM supplemented 1% Penicillin/Streptomycin (Life Technologies, Darmstadt, Germany). After 1 h, monocyte-enriched cells were washed with PBS and cultivated for 13 days in α-MEM supplemented with 10% heat-inactivated fetal calf serum (Biochrom AG, Berlin, Germany), 1% penicillin/streptomycin (Life Technologies, Darmstadt, Germany), 10 ng/mL MCSF, 1 ng/mL RANKL and TGFβ (all Peprotech, Hamburg, Germany). Addition of these three cytokines has been shown to be an effective method to obtain osteoclasts with resorbing activity from human cells [14,15,16]. Medium was replaced every third day. Visual inspection confirmed the continued adherence of cells with each change of medium.

### 3.3. Staining of Osteoclasts

Osteoclast differentiation was evaluated by staining cells for Tartrate-resistant Acid Phosphatase (TRAP) using a Leukocyte Acid Phosphatase Kit (Sigma-Aldrich, Taufkirchen, Germany) according to the manufacturer’s instructions. Images of differentiated osteoclasts were randomly acquired from triplicate wells and differentiated osteoclasts (defined as TRAP positive and multinucleate with ≥3 nuclei) were counted. 

### 3.4. RNA Isolation and Quantitative Real-Time PCR

Total RNA was extracted from osteoclasts growing in 6 well plates (seeding density 3 × 10^6^ cells/mL) using QIAzol Lysis Reagent (Qiagen, Hilden, Germany) and complimentary DNA prepared using the First Strand cDNA Synthesis Kit (Fisher Scientific, Schwerte, Germany). cDNA quantification was carried out using ABsolute quantitative PCR SYBR Green ROX Mix (Fisher Scientific, Schwerte, Germany) on an Applied Biosystems Viia7 real-time PCR system. The relative quantification of cDNA was based on the comparative *C*_t_ method, with two housekeeping gene controls, and normalized to the mean Δ*C*_t_ obtained from patients without mycophenolate mofetil treatment.

The following primers were used for qPCR analysis:

*IMPDH1* 5′-GAGCCCGAGAGCCCTAGATT-3′ & 5′-TCAGGTAGTCCGCCATGCTA-3′;
*IMPDH2* 5′-TGGAGGCAATGTGGTCACTG-3′ & 5′-AGCAATGACCGGAACACCAA-3′;
*RANK* 5′-ACCCTGGACCAACTGTACCT-3′ & 5′- CGGGCAAGTAAACATGGGGT-3′;
*GAPDH* 5′-CATGAGAAGTATGACAACAGCCT-3′ & 5′-AGTCCTTCCACGATACCAAAGT-3′;
*RPL27* 5′-ATCGCCAAGAGATCAAAGATAA-3′ & 5′-TCTGAAGACATCCTTATTGACG-3′.

### 3.5. ELISA for Interferon α and Interferon-γ

For quantitative measurement of serum interferon-α and interferon-γ, ELISA kits from Uscn Life Science Inc. (Wuhan, China) were used according the manufacturer’s instruction. Serum samples were stored at −20 °C until measurement.

### 3.6. Statistical Analysis

Statistical analyses were performed using *GraphPad Prism Software V5* (GraphPad Software Inc., La Jolla, CA, USA) and *SPSS Statistics V21* (SPSS Inc., Standford, CA, USA). Odds ratios were calculated by Fisher’s exact test. *p* Values < 0.05 were considered significant.

## 4. Conclusions

In our *ex vivo* approach we have identified a subgroup of SLE patients in whom osteoclastogensis appears impaired compared to the remaining SLE patients and to healthy individuals. Interestingly this osteoclast differentiation defect correlated inversely with mycophenolate mofetile (MMF) administration and interferon α levels. Further work is required to decipher the underlying mechanisms and to find out if this phenomenon is observed both in *in vivo* experimental models and in clinical observational cohorts and therefore whether it is of critical clinical importance. Since patients without MMF treatment, have higher interferon α serum levels, compared to patients administered with MMF, we conclude that interferon α might contribute to impaired osteoclast phenotype in these SLE patients.
